# The *Aedes aegypti* RNA interference response against Zika virus in the context of co-infection with dengue and chikungunya viruses

**DOI:** 10.1371/journal.pntd.0011456

**Published:** 2023-07-13

**Authors:** Mayke Leggewie, Christina Scherer, Mine Altinli, Rommel J. Gestuveo, Vattipally B. Sreenu, Janina Fuss, Marie Vazeille, Laurence Mousson, Marlis Badusche, Alain Kohl, Anna-Bella Failloux, Esther Schnettler

**Affiliations:** 1 Bernhard-Nocht Institute for Tropical Medicine, Hamburg, Germany; 2 German Center for Infection; Research (DZIF), partner site Hamburg-Luebeck-Borstel-Riems, Germany; 3 MRC-University of Glasgow Centre for Virus Research, Glasgow, United Kingdom; 4 Division of Biological Sciences, University of the Philippines Visayas, Miagao, Iloilo, Philippines; 5 Institute of Clinical Molecular Biology (IKMB), Kiel University, Kiel, Germany; 6 Institut Pasteur, Université Paris Cité, Arboviruses and Insect Vectors, Paris, France; 7 University Hamburg, Faculty of Mathematics, Informatics and Natural Sciences, Hamburg, Germany; Medizinische Universitat Wien, AUSTRIA

## Abstract

Since its detection in 2015 in Brazil, Zika virus (ZIKV) has remained in the spotlight of international public health and research as an emerging arboviral pathogen. In addition to single infection, ZIKV may occur in co-infection with dengue (DENV) and chikungunya (CHIKV) viruses, with whom ZIKV shares geographic distribution and the mosquito *Aedes aegypti* as a vector. The main mosquito immune response against arboviruses is RNA interference (RNAi). It is unknown whether or not the dynamics of the RNAi response differ between single arboviral infections and co-infections. In this study, we investigated the interaction of ZIKV and DENV, as well as ZIKV and CHIKV co-infections with the RNAi response in *Ae*. *aegypti*. Using small RNA sequencing, we found that the efficiency of small RNA production against ZIKV -a hallmark of antiviral RNAi—was mostly similar when comparing single and co-infections with either DENV or CHIKV. Silencing of key antiviral RNAi proteins, showed no change in effect on ZIKV replication when the cell is co-infected with ZIKV and DENV or CHIKV. Interestingly, we observed a negative effect on ZIKV replication during CHIKV co-infection in the context of Ago2-knockout cells, though his effect was absent during DENV co-infection. Overall, this study provides evidence that ZIKV single or co-infections with CHIKV or DENV are equally controlled by RNAi responses. Thus, *Ae*. *aegypti* mosquitoes and derived cells support co-infections of ZIKV with either CHIKV or DENV to a similar level than single infections, as long as the RNAi response is functional.

## Author summary

Zika virus (ZIKV) is a mosquito-borne human-pathogenic arbovirus of the *Flaviviridae* family, genus *Flavivirus*. Other arboviruses, including dengue (DENV) or chikungunya (CHIKV) virus, can occur in the same regions as ZIKV and are also transmitted by *Aedes aegypti*. Notably, it has been shown that these viruses can co-infect this mosquito, andco-transmission occurs. Such processes may add to the serious public health issues already linked to those pathogens. Arbovirus infections in mosquitoes are controlled through an immune response called RNA interference (RNAi). It is however unknown whether immune responses changs when a mosquito is exposed to a co-infection of ZIKV with either DENV or CHIKV. In this study, we provide evidence that ZIKV co-infections with CHIKV or DENV are similarly well controlled by RNAi as single infections. These findings give new insights into the dynamics of arboviral co-infections in mosquito vectors that increase our understanding of co-infection scenarios during arbovirus outbreaks.

## 1. Introduction

Zika virus (ZIKV) is an arbovirus, of the family *Flaviviridae* (genus *Flavivirus*). Following its detection in the Americas in 2015 and the association with Guillain-Barré-syndrome and microcephaly, ZIKV has remained in the international public health and research spotlight [1,2]. In addition to ZIKV, other important arboviruses of public health concern, such as dengue virus (DENV)—a related flavivirus—and chikungunya virus (CHIKV)—an alphavirus–are known to circulate in the same geographical areas [3]. Besides, various studies of human cases showed that ZIKV can also occur in co-infection with DENV [4–8], CHIKV [5,7–9] or both [4,8,10] and that these co-infections seem to be common in both endemic and epidemic regions, thus increasing the public health burden. There is a notable lack of studies assessing the impact of arboviral co-infection within the patient in terms of short- and/or long-term clinical outcomes [11]. The interaction of the viruses is likely to be complex and the outcome is expected to vary, perhaps even on a case-to-case basis. It is to note however that the enhancement of disease severity might occur when the arboviruses support each other’s infection or have an exacerbating effect on the immune response of the host [11]. Reports of CHIKV, DENV and ZIKV sharing cell tropism and mechanisms of host immune response interference strengthen the notion that enhancement of disease severity is possible for a co-infection with these viruses [11]. Due to the lack of effective drugs and vaccines against arboviruses, control relies on the prevention of disease, i.e., vector control [12]. ZIKV, CHIKV and DENV are transmitted by members of the *Aedes* genus, with *Aedes aegypti* mosquitoes shown to be one of the major vectors for all three viruses [13–15]. Therefore, co-infections and subsequent co-transmission by the same mosquitoes could occur. Indeed, co-infection studies in *Ae*. *aegypti* mosquitoes have revealed that these mosquitoes can be infected with and transmit all combinations of ZIKV, CHIKV and DENV infection simultaneously [13,16–18]. Interestingly, co-infections do not seem to affect mosquito susceptibility and vector competence, except for CHIKV [13].

The main mosquito immune response against arbovirus infections in mosquitoes is RNA interference (RNAi), which might also have a role in differentially regulating infection dynamics during co-infections. In *Ae*. *aegypti*, the main RNAi pathways involved in the control of arboviral infections are the exogenous small interfering RNA (exo-siRNA) and the P-element-induced wimpy testis (PIWI)-interacting RNA (piRNA) pathways [19].

The antiviral exo-siRNA pathway is initiated by the presence of viral double-stranded (ds) RNA derived from viral replication. Once detected, the dsRNA is cut by the enzyme Dicer 2 (Dcr2) into 21-nucleotide (nt) sized viral-specific (v)siRNAs. These are then incorporated into the RNA-induced silencing complex (RISC), where they associate with the Argonaute 2 (Ago2) protein, which uses a single strand of the vsiRNA as a guide to locate and target complementary viral RNA sequences within the cell. The viral RNA is subsequently cleaved by the complex, resulting in the inhibition of viral replication [19,20]. The exo-siRNA pathway is involved in the control of all major arboviruses, as shown by the isolation of vsiRNAs from infected mosquitoes and derived cells [21]. Knock-down (KD) or knock-out (KO) of key players of the exo-siRNA pathway, including Dcr2 and Ago2, was shown to result in increase in viral replication for most tested arboviruses [22–27]. An exemption is ZIKV, where silencing or knock-out of Ago2 had no significant antiviral effect, in contrast to Dcr2 knock-outs [28,29].

piRNAs are small RNAs with a relatively broad size range of 24–30 nt. Arbovirus-derived piRNAs have been described in both mosquitoes and mosquito-derived cells [22–25,29–39]. Key players in the biogenesis of virus-derived piRNAs in *Ae*. *aegypti*-derived Aag2 cells were identified as Piwi5/Piwi6 and Ago3. RNA transcripts are bound by either Piwi5/6 or Ago3 and feed into the so-called ping-pong amplification cycle. The piRNA molecules produced by the ping-pong amplification cycle are further characterized by a distinct nucleotide bias for either uridine at position 1 (U1; antisense sequence) or adenine at position 10 (A10; sense sequence) as well as a sequence overlap of 10 nucleotides [29,32,34,39]. Another Piwi protein, Piwi4, is not directly involved in the biogenesis of ping-pong cycle piRNAs [24,31,33]. However it binds DENV-specific piRNAs derived from viral cDNA in *Ae*. *aegypti* [24], interacts with proteins of the piRNA and siRNA pathways [24,31,40] and is antiviral for all tested arboviruses [22–24,29,31–33].

RNAi is involved in the control of ZIKV [29], DENV [24,25,38,39] and CHIKV [32,36,37,41] infections of *Ae*. *aegypti-*derived cells. However, it is unknown whether the dynamics of the mosquito immune system differ between single arboviral infections and co-infections. Here, we investigated the interactions of ZIKV-CHIKV or ZIKV-DENV co-infections with the RNAi response in *Ae*. *aegypti*-derived cells and mosquitoes. We confirm previous reports that co-infections are well tolerated by mosquitoes. The RNAi response to ZIKV-CHIKV co-infections *in vitro* resembled previous results from single infections with the individual viruses. To understand, whether mosquito RNAi responses regulate arbovirus co-infections in mosquitoes, we silenced/ knocked out RNAi response effectors in mosquito cells. The same antiviral RNAi proteins, resulting in increased virus infection in case of silencing/ knock outs were identified in case of single and co-infections for the corresponding viruses. However, differences in the effects of antiviral RNAi proteins were observed in a virus-dependent manner. Piwi4 was antiviral for ZIKV. Ago2 antiviral activity was observed for CHIKV, but not for ZIKV. In contrast to previous reports, we found that silencing or knock-out of any of the selected RNAi proteins revealed no antiviral activity against DENV-1, neither in the single or co- infection with ZIKV.

Taken together, mosquitoes and derived cells support co-infections of ZIKV with either CHIKV or DENV to a similar level than single infections, when the RNAi response is functional.

## 2. Methods

### Cell lines and mosquitoes

Aag2-AF5 cells (obtained from European Collection of Cell Cultures (ECACC); 19022601; called AF5) are a single clone cell line of *Ae*. *aegypti*-derived Aag2 cells [42]. Aag2-AF525 (AF525) is an Ago2 knock-out line derived from abovementioned Aag2-AF5 cells [28]. Mosquito-derived cell lines were grown in Leibovitz’s L-15 medium (Thermo Fisher Scientific, USA) supplemented with 10% fetal calf serum (FCS) (GIBCO Thermo Fisher Scientific, USA), penicillin-streptomycin (P/S, final concentration 100 units/ml and 100 μg/ml, respectively) (Thermo Fisher Scientific, USA) and 10% Tryptose Phosphate broth (TPB) (GIBCO Thermo Fisher Scientifc, USA) at 28°C.

A549/BVDV-NPro (A549 Npro) cells, which are stably expressing the bovine viral diarrhea virus NPro protein (provided by R.E. Randall, University of St. Andrews, UK) [43] and Vero cells (Vero81; ATCC CCL-81, *Cercopithecus aethiops*) were maintained in Dulbecco’s modified Eagle’s medium (DMEM) (PAN Biotech, Germany). Medium was supplemented with 10% (A549 NPro) or 5% (Vero81) FCS, 10% TPB and P/S. Cells were grown at 37°C / 5% CO_2_.

*Ae*. *aegypti* Urca (collected in 2016 Rio de Janeiro using ovitraps; [44]) mosquitoes strain were maintained on a 10% sucrose solution at 27–28°C with a photophase of 12h and around 80% relative humidity.

### Virus stocks

For silencing and knock-out experiments of key RNAi proteins, the following viruses were used. The Brazilian ZIKV strain PE243 has been already described elsewhere [45]. CHIKV (001V-02242; strain UVE/CHIKV/2014/FR/CNR_24) and the DENV-1 (001V-02228; strain UVE/DENV-1/2014/FR/CNR_25329) were provided by the European Virus Archive–GLOBAL (EVAg) by Aix-Marseille University (AMU). ZIKV and DENV-1 stocks were produced on A549 NPro cells. For the production of CHIKV stocks, Vero81 cells were used. Final stocks were titrated by 50% tissue culture infective dose (TCID_50_) using either Vero81 cells (CHIKV and ZIKV) or A549 NPro cells (DENV).

The following virus stocks were used for the small RNA sequencing analysis: DENV-1 strain DENV-1/MX/BID-V7614/2009 (KJ189345), ZIKV strain MRS_OPY_Martinique_PaRi_2015 (KU647676) and CHIKV strain 06–021 (AM258992).

### dsRNA synthesis

*In vitro*-transcribed dsRNA for *Ae*. *aegypti* Ago2, Ago3, Piwi4, Piwi5 and Piwi6 as well as eGFP were produced via T7 RNA polymerase transcription using PCR amplified fragments as previously described [33]. In short, gene-specific fragments, flanked by T7 RNA polymerase promoter sequences, were amplified by PCR. All PCR-amplified fragments were validated by Sanger sequencing and used for *in vitro* transcription. Subsequent column-based purification using the MEGAscript RNAi kit (Thermo Fisher Scientific, USA) was performed according to manufacturer’s instructions.

### Small RNA sequencing and analysis

To investigate the production of virus specific small RNAs during co-infection and single infection, *Ae*. *aegypti* mosquitoes were infected with either ZIKV or co-infected with a mix of DENV+ZIKV or CHIKV+ZIKV (10^7^ FFU/ml concentration of each virus) [46]. Total RNA (of pooled and unpooled mosquito samples; see details in Table B in S1 File) was isolated at 14 days post infection (dpi) with TRIzol LS (Invitrogen, USA) according to manufacturer’s instructions. 1 μg total RNA was used for small RNA sequencing by a BGISEQ-500 at BGI Tech (Hong Kong, China) as previously described [28].

In addition, the production of virus specific small RNAs during both co-infection and single infection was investigated *in vitro* using AF5 cells. For this, 8x10^5^ cells were seeded into a 6-well plate and infected with either ZIKV (MOI 1) or co-infected with a mix of DENV+ZIKV or CHIKV+ZIKV (MOI 1 per virus). Total RNA was isolated at 96 hpi with TRIzol (Invitrogen, USA), according to the manufacturer’s instructions. Total RNA was sequenced at CCGA (Kiel, Germany) using 100 ng total RNA for library preparation with the Nextflex small RNA-Seq kit v3 (PerkinElmer Inc., USA), followed by library sequencing on the NovaSeq6000 SP v1.0 platform.

Analysis of small RNAs was performed as previously described [33]. Reads mapping to the genome or anti-genome of ZIKV were expressed as % mapped reads or reads per million (RPM) to normalize the data allowing comparison between single and co-infection experiments.

For small RNA analysis of mosquito samples (ZIKV: 2 repeats, ZIKV+CHIKV: 4 repeats and ZIKV+DENV: 5 repeats) and cells (ZIKV: 2 repeats, ZIKV+CHIKV: 2 repeats and ZIKV+DENV: 1), data from independent samples were first separately analyzed; following this the means of virus specific small RNAs were determined.

To determine the relationship between the ZIKV-derived siRNAs during single vs. co-infection experiments, a linear regression analysis was performed between the mean reads in RPM from ZIKV alone (x-axis) and ZIKV+CHIKV or ZIKV+DENV (y-axis) plotted with respect to nt position. The r-squared values reflect the degree of variability of ZIKV-derived siRNAs mapped to the genome or anti-genome as an outcome of single or co-infection. In addition, to reveal patterns in the abundance of the ZIKV-derived siRNAs during co-infection with CHIKV or DENV, the mean reads were transformed into fold change in log2 (ZIKV+CHIKV or ZIKV+DENV relative to ZIKV alone) per nt position (genome or anti-genome) and visualized as a scatter plot.

#### Searching for regions of viral genome similarity of co-infecting viruses

To investigate if during co-infection experiments, vsiRNA from one virus could theoretically target the co-infecting virus, the GenBank database was used to obtain the genome sequences for the DENV type 1 (GenBank id: KJ189345), ZIKV strain MRS_OPY_Martinique_PaRi_2015 (GenBank id: KU647676), and CHIKV (GenBank id: AM258992). Sequences were divided using the splitter programme from the EMBOSS software (version 6.6.0.0) into 21-mer (k-mers with k = 21) sequences with 20 nt overlap. With the preset settings for blastn, each k-mer set was compared to viral genomes. Sequences with at least 20 nucleotide identities that permit one difference were chosen as matches.

### Knock-down studies

AF5 cells were seeded in 24-well plates, with 2x10^5^ cells/well. The following day, cells were transfected with 200 ng gene-specific dsRNA or control dsRNA (eGFP) per well using 1 μl of Dharmafect2 reagent (GE Dharmacon). At 24 hours post transfection (hpt), either single infections with ZIKV, DENV or CHIKV (MOI 1) or co-infections in the combinations of ZIKV+CHIKV or ZIKV+DENV (MOI 1 per virus) were performed in technical duplicates. At 96 hours post infection (hpi), the technical duplicates were pooled, and RNA was isolated from cells using TRIzol (Invitrogen, USA). 1.5 μg RNA was used to produce cDNA using Moloney murine leukemia virus (M-MLV) reverse transcriptase (Promega, USA) and random hexamer primers (Thermo Fisher Scientific, USA). A SYBR green qPCR (QIAGEN, Germany) was performed for viral targets using gene-specific primers (Table A in S1 File). qPCR data were analysed using the 2^-ΔΔCT^ method with ribosomal protein S7 RNA as the housekeeping gene, and eGFP dsRNA samples as the control group. Overall, each experiment was independently repeated three times.

### Virus infections of knock-out cell lines

AF5 and AF525 cells were seeded in 24-well plates at 2x10^5^ cells/well. The following day, cells were infected with either ZIKV, DENV or CHIKV individually (MOI 1) or with a combination of either ZIKV+CHIKV or ZIKV+DENV (MOI 1 per virus). All infection scenarios were set up in duplicates. At 96 hpi, duplicates were pooled, and RNA was isolated from cells using TRIzol (Invitrogen, USA). RNA was further processed as described for the knock-down experiments above. Notably, in this case, AF5 cells were used as the control group. Each experiment was independently repeated three times.

### Statistical analysis

All statistical analyses and data visualization were run in R version 4.1.2 except for the linear association and scatter plot analyses which were performed in GraphPad Prism v.7. Normally distributed data were analysed with ANOVA or pairwise-t-test. Not normally distributed data were analysed using Kruskall-Wallis (KW) and Dunn’s post-hoc test. P values for all multiple comparisons were corrected using Bonferroni correction. P value <0.05 is considered as statistically significant. Results of the statistical analysis are combined in Table C, D and F in S1 File.

## 3. Results

### Small RNA production profiles of ZIKV during single or co-infection with CHIKV or DENV, in vitro and in vivo

To compare the virus-specific small RNA production between co-infection and single infection, *Ae*. *aegypti* mosquitoes were infected either with only ZIKV or co-infected (ZIKV+CHIKV, ZIKV+DENV) by bloodmeal. Successful infection was verified by qPCR after 14 days and total RNA isolated (Table B in S1 File). Following this, small RNAs were sequenced, mapped to the viral genomes and analysed (Table B in S1 File).

**Fig 1:**
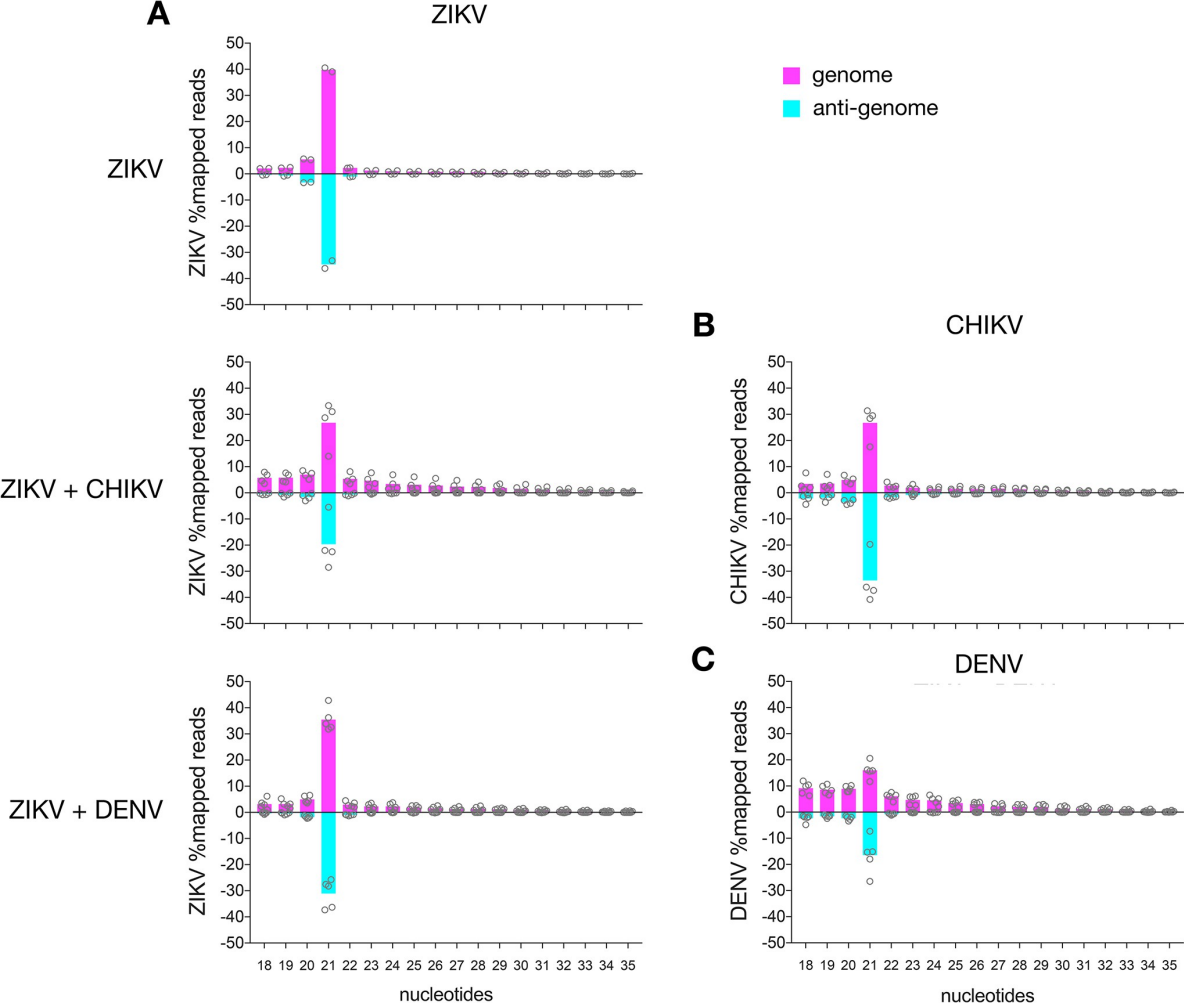
Virus-specific small RNA production in infected Ae. aegypti mosquitoes. Size distribution of small RNAs mapping to ZIKV (A), either single or in co-infection with CHIKV (B) or DENV (C) in infected Ae. aegypti mosquitoes (14 dpi). Sequences mapping to viral genome (pink) and antigenome (blue). The y axis shows the proportion of small RNAs of a given length to the total virus-specific small RNA reads. In addition to the separated data of the independent repeats (shown as circles), the means of independent repeats is shown (ZIKV: 2 repeats, ZIKV+CHIKV: 4 repeats and ZIKV+DENV: 5 repeats).

**Fig 2:**
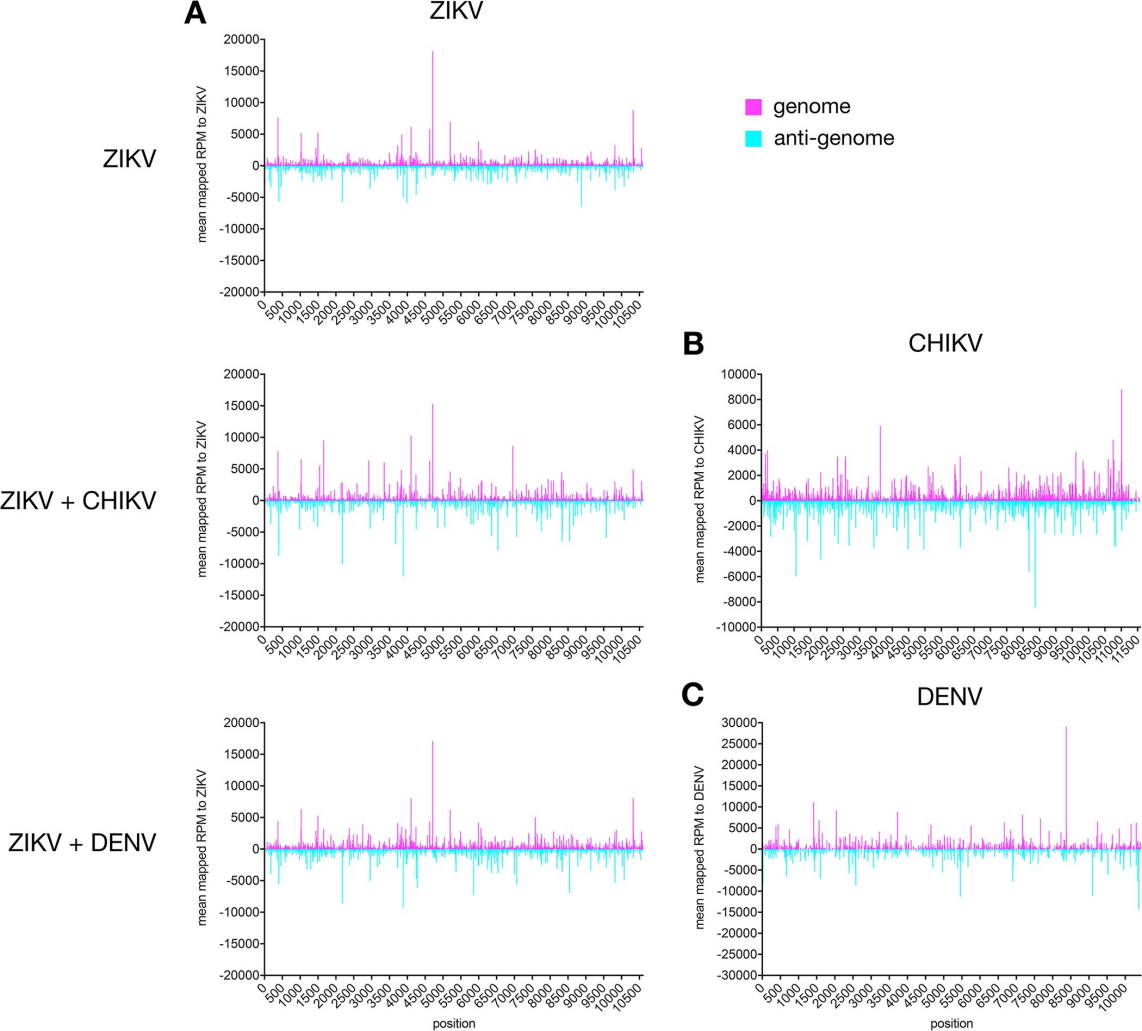
Mapping of 21 nt vsiRNAs small RNAs in Ae. aegypti mosquitoes. Distribution of 21 nt long small RNAs in ZIKV (A), either single or in co-infection with CHIKV (B) or DENV (C) in infected Ae. aegypti mosquitoes (14 dpi). The y axis shows the mean (of several independent repeats) mapped reads per million (RPM) to the viral genome (pink) and anti-genome (blue). Means of independent repeats are shown (ZIKV: 2 repeats, ZIKV+CHIKV: 4 repeats and ZIKV+DENV: 5 repeats).

In all samples, the majority of virus specific small RNAs were 21 nt in length and mapped mostly, and to a similar extent to the genome and the anti-genome (Fig 1), with no real emphasis or obvious preference for specific regions (Fig 2). Notably, less ZIKV-specific 21 nt vsiRNAs were observed during co-infections with CHIKV, compared to single ZIKV infection or co-infection with DENV (Fig 1A and TableB in S1 File). We investigated the linear relationship between the mapped reads of the single ZIKV infection versus the co-infection. ZIKV-specific siRNA mapped to the genome displayed a higher positive relationship compared to the ZIKV-specific siRNA mapped to the anti-genome (Figs 1A and A in S1 File).

The scatter plot looking at the relative expression of the reads mapped to ZIKV during single and co-infections, showed a strong dispersion of reads mapping to ZIKV anti-genome with increases in mapped reads in co-infections with both CHIKV and DENV. These analyses support the differences in dispersion of vsiRNAs mapping to the anti-genome of ZIKV during co-infections, especially with CHIKV (Figs 1A and A in S1 File). They also support the marked differences in anti-genome mapping of ZIKV-derived vsiRNAs during co-infection. Following co-infection of ZIKV and DENV, relatively low amounts of vsiRNA were derived from DENV when compared to ZIKV (Fig 1A and 1C).

Overall, low numbers of reads mapped to the size of vpiRNAs (25–29 nts) (Figs 1, and B, C and TableB in S1 File). Only for CHIKV, vpiRNAs with ping-pong production specific characteristics were observed (Fig C in S1 File). This was not observed for ZIKV nor DENV, regardless of single or co-infections.

To understand if these differences in virus specific small RNAs between single and co-infections could also happen in mosquito cells, infections were performed in cell culture. *Ae*. *aegypti*-derived AF5 cells were either single infected (ZIKV) or co-infected (ZIKV+CHIKV and ZIKV+DENV), total RNA isolated, small RNA sequenced, mapped and analyzed (TableB in S1 File).

**Fig 3:**
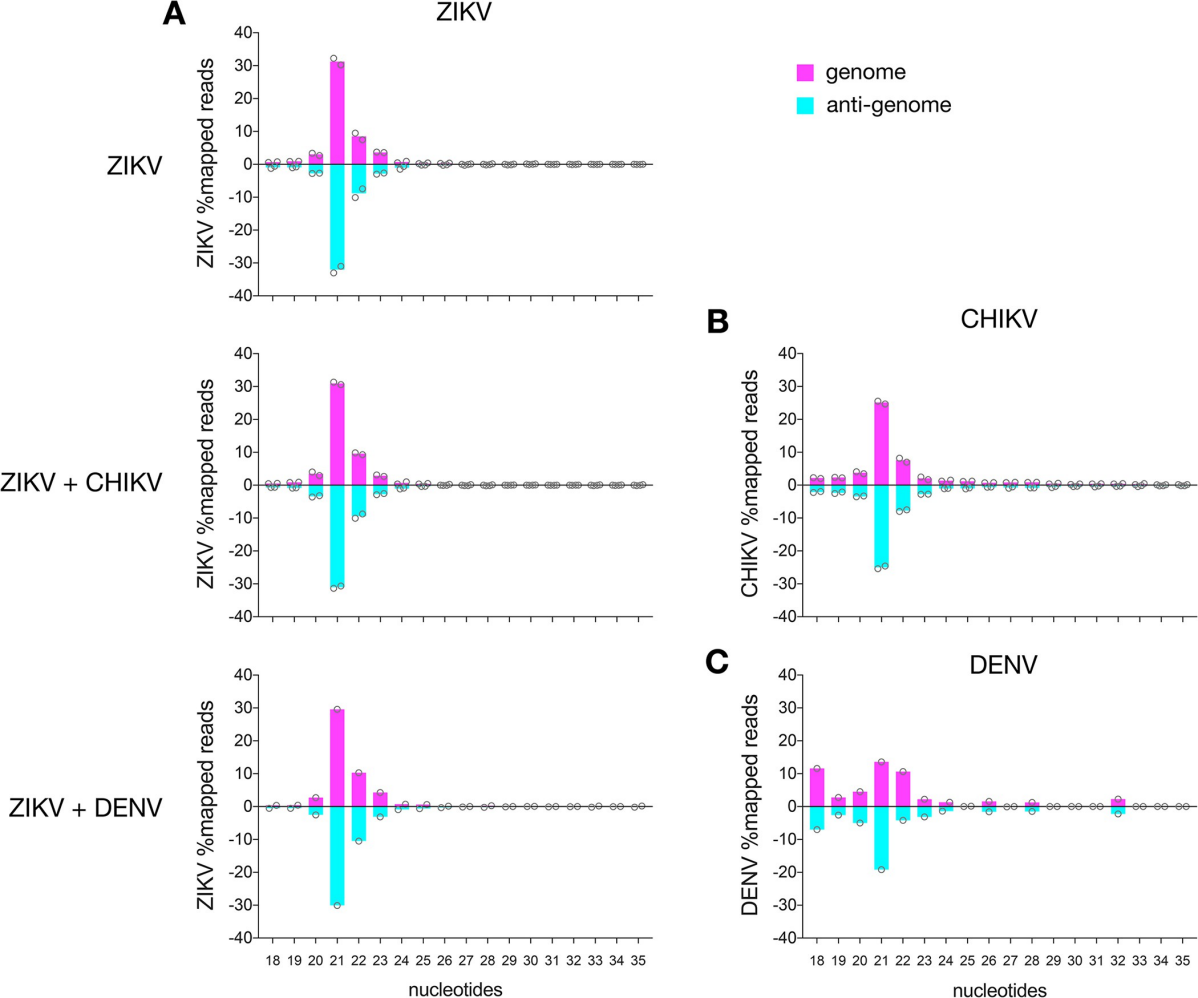
Virus-specific small RNA production in Ae. aegypti-derived AF5 cells. Size distribution of small RNAs from ZIKV (A), either single or in co-infection with CHIKV (B) or DENV (C) in infected cells (96 hpi). Sequences mapping to the viral (ZIKV, CHIKV and DENV) genome (pink) and anti-genome (blue). In addition to the individual data of independent repeats (shown as circles), the means are shown (ZIKV: 2 repeats, ZIKV+CHIKV: 2 repeats and ZIKV+DENV: 1).

**Fig 4:**
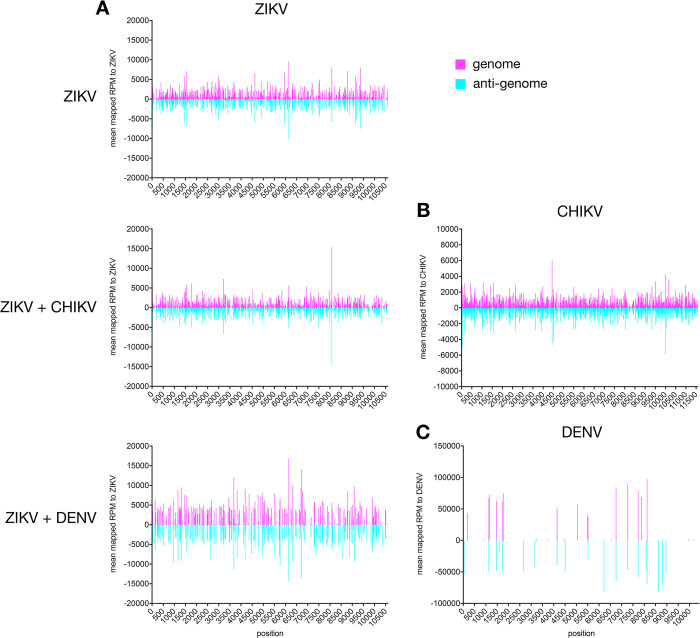
Mapping of 21 nt vsiRNAs in infected Ae. aegpyti-derived AF5 cells. Distribution of 21 nt vsiRNAs in ZIKV (A), either single or in co-infection with CHIKV (B) or DENV (C) in infected cells (96 hpi). The y axis shows the mapped reads per million (RPM) to the respective viral genome and anti-genome. The means of independent repeats are shown (ZIKV: 2 repeats, ZIKV+CHIKV: 2 repeats and ZIKV+DENV: 1).

ZIKV-specific small RNAs were mostly 21 nt in length and derived from the genome and the anti-genome (Fig 3A). The amounts of ZIKV-specific siRNAs between single and co-infections in the AF5 cells were very similar (Figs 3A, 4A and D in S1 File). The data obtained for virus-specific piRNAs produced in AF5 cells were comparable to the results obtained in mosquitoes, although the amount of CHIKV-specific piRNAs (Fig E in S1 File) and sequence signatures were not as obvious (Fig F in S1 File). This is probably due to a generally lower amount of small RNAs in the size range of piRNAs in these samples, due to a different library/ sequencing method. Similar decreases in piRNA sized small RNAs were previously observed for these sequencing protocols [28]. Small RNA mapping and distribution along the genome/ antigenome, specifically vsiRNAs of CHIKV were similar in cells compared to the infected mosquitoes (Figs 2B and 4B). In case of DENV specific small RNAs, the amount of vsiRNAs produced in infected cells was very low and therefore, no good mapping along the genome and anti-genome was possible (Fig 4C).

To investigate if vsiRNAs derived from one virus could target the co-infecting virus, in silico analysis was performed. The ZIKV genome was divided in 21mers and analysed for complementarities to either the CHIKV or DENV genome allowing none or 1 mismatch. In case of ZIKV and CHIKV, no perfect complementary pairs were identified that could potentially cross target the co-infecting viruses. In case of ZIKV and DENV, a small number of high similarity pairs (allowing 1 mismatch) were identified. One pair matched to a region in the envelope protein and the other 3 pair matched to different regions of the NS5 (Table E in S1 File).

### Effects of silencing or knock out of RNAi effectors on arbovirus single versus co-infection in Ae. aegypti-derived cells

#### dsRNA-based silencing of RNAi effectors

Antiviral activities of RNAi effector proteins had only been investigated for single arbovirus infections. Nothing was known if the interplay of these proteins and their ability to act antivirally is the same during co-infection and single arbovirus infections. To investigate which RNAi effector(s) acted antivirally during co-infection, dsRNA-based silencing experiments were performed in *Ae*. *aegypti*-derived AF5 cells followed by single (ZIKV, CHIKV or DENV) or co-infection (ZIKV+CHIKV or ZIKV+DENV). Cells were transfected with specific dsRNAs (targeting Ago2, Ago3, Piwi4, Piwi5, Piwi6 and eGFP as control) and 24 hours later infected at MOI 1. Total RNA was isolated at 96 hpi and successful silencing of the target transcripts verified by qPCR, using dseGFP transfected cells as control. Viral RNA levels were determined by qPCR and effects on virus infection compared to dseGFP control cells between single and co-infections.

**Fig 5:**
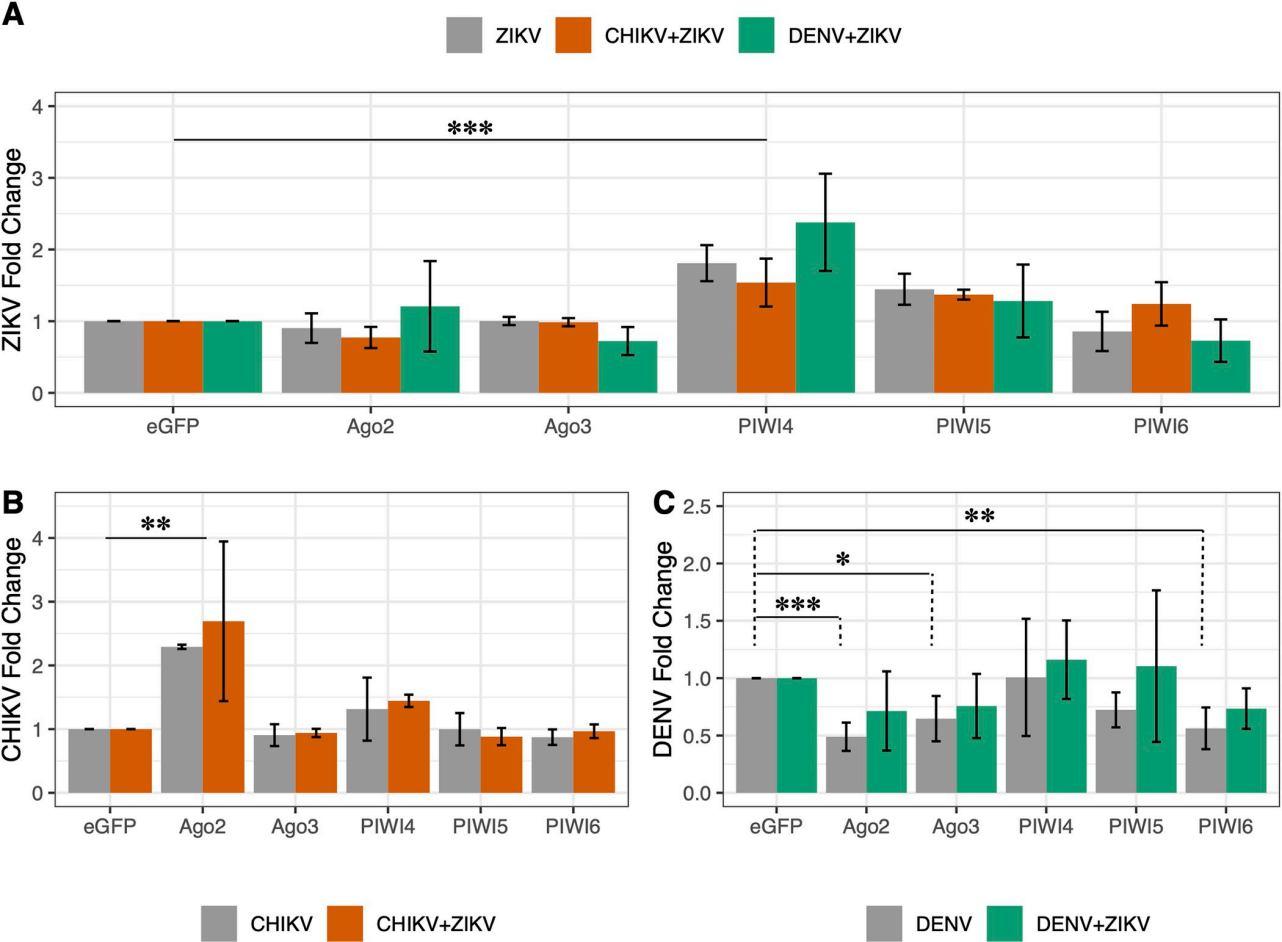
Effect of silencing of RNAi-related proteins on arbovirus (co-)infection. AF5 cell were transfected with gene-specific or control (eGFP) dsRNA, followed by single (DENV, CHIKV or ZIKV) or co- infection (DENV+ZIKV or CHIKV+ZIKV). Total RNA was isolated at 96 hpi and viral RNA quantified using virus-specific primers (A: ZIKV, B: CHIKV, C: DENV) with ribosomal protein S7 RNA as housekeeping transcript. Viral RNA fold changes were calculated using the 2^-ΔΔCT^ method. Bar plots represent the mean fold changes calculated for each group with error bars. Data shown are from either three (ZIKV, CHIKV, ZIKV+CHIKV) or six (DENV, ZIKV+DENV) independent experiments (*: p < 0.05; **: p < 0.01, ***: p <0.001).

The effects of silencing of RNAi effectors, on ZIKV replication was generally not influenced by co-infections (Fig 5A). More specifically, ZIKV replication was not significantly influenced by silencing Ago2, Piwi5 or Piwi6 irrespective of the type of infection. ZIKV replication increased significantly only when Piwi4 was silenced (Fig 5A). Antiviral activities of RNAi effector proteins against CHIKV were the same for single and co-infections (Fig 5C). In both cases, CHIKV replication increased significantly only in Ago2 silenced cells.

For DENV-1, both infection type and silencing had significant effects on viral replication (Fig 5B). Interestingly, Ago2, Ago3 and Piwi 6 silencing resulted in a significant decrease of DENV-1 replication during single infection, but this was not the case in the DENV+ZIKV co-infected cells (Fig 5B).

#### Arbovirus infections in Ago2-knock out Ae. aegypti-derived cells

To further understand the role of the antiviral RNAi response, experiments were repeated in knock-out cells. For this Ago2 knock-out cells (AF525) were used. AF525 or AF5 (control) cells were either single or co-infected (ZIKV+CHIKV, ZIKV+DENV) at MOI 1 and total RNA isolated at 96 hpi. Viral RNA levels were determined by qPCR and compared between single and co-infected samples as well as between AF525 and AF5 cells.

**Fig 6:**
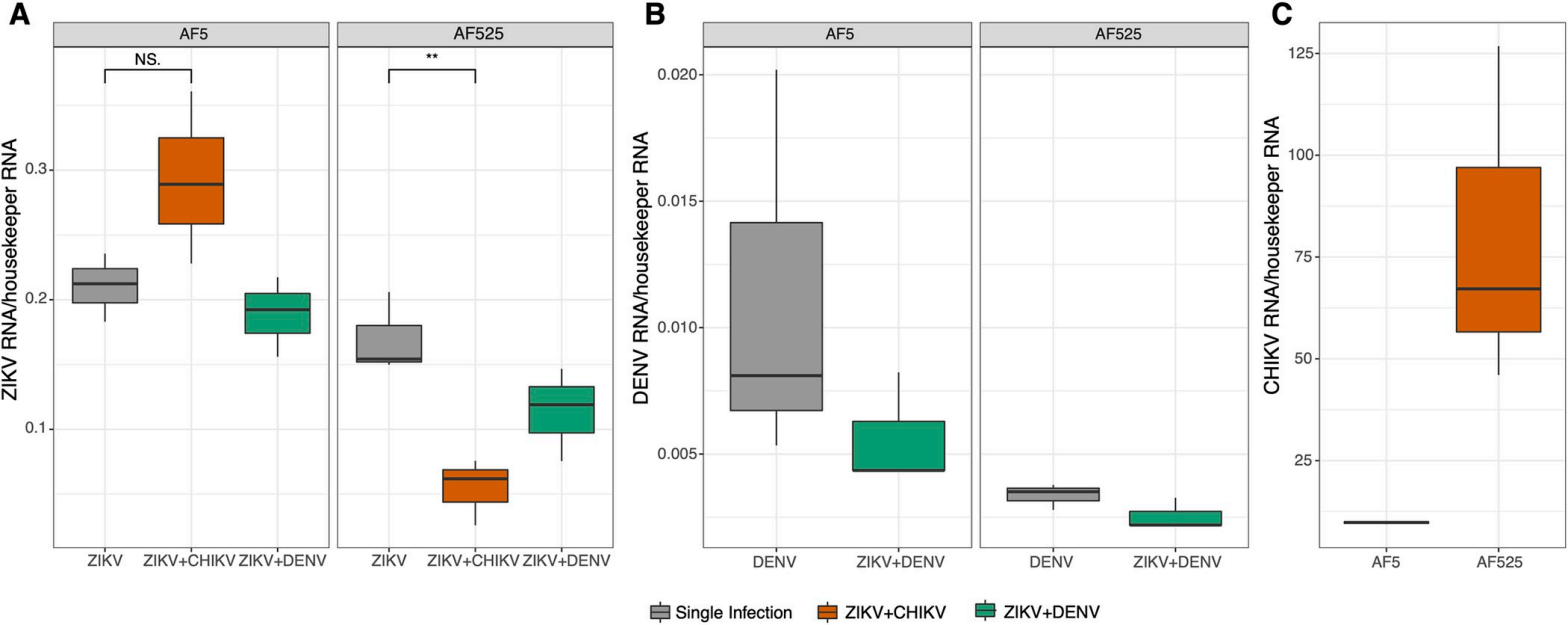
Arbovirus replication in Ago2 knock out cells during single and co-infections. Ae. aegypti-derived AF525 (Ago2 deficient) or parental AF5 cells were infected either with ZIKV, CHIKV or DENV (grey) or co-infected (ZIKV+DENV: green or ZIKV+CHIKV: orange) (MOI1). Viral RNA levels (A: ZIKV, B: DENV, C: CHIKV) at 96 hpi were determined by qPCR and normalized to ribosomal S7 gene. Data shown are the median with min and max values from three independent experiments. **, p<0.01.

Overall, ZIKV RNA levels were lower in AF525 compared to AF5 cells (Fig 6A); however single infection of ZIKV resulted in similar ZIKV RNA level in AF5 and AF525 cells. In contrast, ZIKV RNA level were significantly lower in AF525 cells compared to AF5 cells in case of co-infection with CHIKV or DENV (Fig 6 and Table D in S1 File) Comparing the different infection scenarios in AF525 cells, the strongest decrease of ZIKV was observed during co-infection with CHIKV (Fig 6A). In contrast, CHIKV replication increased in ZIKV co-infected AF525 cells compared to AF5 control cells, although this increase was not statistically significant (Fig 6C). Similar results were observed for CHIKV and ZIKV in AF5 and AF525 cells, using luciferase expressing viruses (Fig F and TableF in S1 File).

Similar to what we observed for ZIKV RNA levels, DENV-1 decreased generally in AF525 cells compared to AF5 cells (Fig 6B). However, this effect on DENV-1 was only significant in co-infection with ZIKV (Table D in S1 File).

## 4. Discussion

Studies investigating the vector competence of *Ae*. *aegypti* co-infected with ZIKV, DENV and CHIKV have shown that co-infection does not have an impact on the competence of the mosquito for any individual virus [13,16]. In fact, transmission rates were similar in single and co-infected individuals [13]. At this time, it is unknown whether the antiviral RNAi response is able to target both co-infecting arboviruses to a similar extent. Here, we investigated the RNAi response against ZIKV in the context of co-infections with DENV or CHIKV. Our results show that ZIKV is targeted, in most cases, as efficient by the RNAi response during co-infection as during a single infection. Overall, virus-specific small RNA production showed similar patterns for ZIKV during a single infection or co-infection, in manner comparable to previous reports [29]. Mostly 21 nt vsiRNAs are produced during ZIKV infection, both in mosquitoes and cells, that were uniformly distributed along the genome and anti-genome. In contrast, vpiRNA-sized small RNAs were produced mostly from the genome and mapped to a small number of distinct sequences.

Similarly to ZIKV, CHIKV as well as DENV-1 specific small RNAs during co-infection with ZIKV were comparable to previously published data, though different virus strains (for CHIKV) or even different serotypes (DENV-1 versus DENV-2) were used [25,37,39]. CHIKV and DENV-1 infections produced mostly 21 nt vsiRNAs mapping across the genome and antigenome. For CHIKV, ping-pong-produced vpiRNAs were produced, mostly mapping to the genome around the 5´end area of the subgenomic RNA. In contrast, DENV resulted in (similar to ZIKV) only a small number of vpiRNA-sized small RNAs, mapping mostly to specific areas of the viral genome.

The ability of the RNAi response to efficiently target arbovirus infection in case of acute co-infection is further supported by silencing experiments that target RNAi effectors. For both ZIKV and CHIKV, antiviral activities of RNAi effectors were observed for both single and co-infection experiments. However, differences were observed regarding the antiviral effectors. Silencing of Piwi4 increased ZIKV replication, corresponding to previous results in Aag2 cells [29], but only showed a non-significant increase for CHIKV. Previous studies have shown a significant increase of CHIKV in Piwi4 silenced cells. The observed discrepancy could be due to differences in the experimental setup, virus strain, MOI (0.01 versus 1), time of detection (48 hpi versus 96 hpi) and detection method (e.g., viral RNA level in cells, infectious virus particle in the supernatant, luciferase protein in the cells) used. Ago2 showed antiviral activity against CHIKV as expected [32,47]. As observed for other alphaviruses [24,28,33,48,49], the RNAi response against CHIKV seems to be centered on the exo-siRNA pathway, with Ago2 silencing generating the strongest viral increase. However, no significant increase of ZIKV replication was observed in response to silencing of Ago2. This observation mirrors previous observations [28,29]. Notably, there is also no change in effect on ZIKV replication during co-infection with DENV or CHIKV in AF5 cells. This indicates that the RNAi response towards ZIKV is unchanged in the context of a co-infection, and that the co-infecting virus does not affect this response, either positively or negatively. This is in line with the *in-silico* data analysing possible cross-targeting effects of vsiRNAs. Even though a small number of 21 nt siRNAs sharing complementarity (with one mismatch) were identified for ZIKV and DENV, no interference could be detected. This is in line with previous findings on Palm Creek virus interactions with arboviruses, where no correlation between the degree of nucleotide similarity and the occurrence of interference was detected [50].

Interestingly, CHIKV negatively affected ZIKV infection during co-infections in case of Ago2 knock outs, but not AF5 cells. In contrast, ZIKV infection was not negatively affected in Ago2 knock-out cells during DENV-1 co-infections. This observation correlates well with the increase of CHIKV in AF525 cells (Fig G in S1 File) and the observed antiviral activity of Ago2 for CHIKV [32]; in contrast to an absence of effect on ZIKV. A similar observation was described *in vivo* in *Ae*. *aegypti* mosquitoes co-infected with ZIKV and CHIKV [13], where CHIKV/ZIKV co-exposure resulted in minimally decreased ZIKV infection rates. The authors hypothesized that this decrease might have been linked to competition effects [13]. It is possible that such an effect is amplified in absence of Ago2, which is highly antiviral against CHIKV.

We have focused on one time point where we expected a well-established viral infection. It is not known how virus-specific small RNA production or RNAi effectors may differ at earlier time points of infection. Previous research has shown that under specific circumstances (e.g. virus combination, time points), differences in infection, transmission and/ or dissemination can be observed in *Ae*. *aegypti* [13,51], but that such differences early in infection do not always translate to differences at later time points. A notable limitation of our study is the focus on the late response (e.g. 14 dpi in mosquitoes and 96 hpi in cells) to viral infection. It is possible that the RNAi response to each virus might differ during establishment of infection, which might impact the final outcome depending on the co-infection phenotype. Earlier time-points should be included in future investigations on the effect of viral co-infection on RNAi.

Silencing results with DENV-1 were found to differ from previous reports on DENV; although DENV-specific small RNAs are very similar [25,38,39]. No increases in DENV-1 replication, in response to silencing of any of the RNAi effectors, were observed. In contrast, previous reports have shown Ago2, Dcr2 and Piwi4 to act antivirally against DENV [24–26]. However, in addition to using a different DENV strain (DENV2 instead of DENV1), these studies also utilized different cell lines or performed experiments *in vivo*. Furthermore, there was no increase in DENV-1 replication in AF525 cells both after single or co-infection with ZIKV. On the contrary, there appeared to be an overall decrease in DENV-1 replication in absence of Ago2, suggesting a proviral effect of this RNAi effector.

It is assumed that a delicate balance between arbovirus and mosquito vector is essential for a successful infection and transmission of arboviruses by mosquitoes. The balance between arbovirus replication and the mosquito immune response ensures that the arbovirus infection is sufficient for transmission without high pathogenicity in the mosquito vector. Our study provides evidence that ZIKV co-infection with DENV or CHIKV are comparable in terms of RNAi responses, to single infections; *Ae*. *aegypti* is able to target one as well as two viruses to a similar extent. Importantly this is true as long as the mosquito (or cells) can mount a functional antiviral RNAi response. If parts of the immune response are dysfunctional, the balance of virus replication of the different viruses can be affected. This specifically applies for RNAi effectors of the immune system that appear to act differently against arboviruses, like Ago2 which acts antiviral against CHIKV but not ZIKV. This needs to be taken into account when immune effectors become targets for intervention strategies.

## Supporting information

S1 File **Fig A.** Analysis of ZIKV-derived vsiRNAs from Ae. aegypti mosquitoes infected with ZIKV and CHIKV or DENV. **Fig B**. Mapping of virus-specific 25–29 nts small RNAs produced in infected mosquitoes, along the corresponding virus genome. **Fig C**. Characterisation of CHIKV-specific piRNA-like small RNAs in Ae. aegypti mosquitoes. **Fig D**. Analysis of ZIKV-derived vsiRNAs from AF5 cells infected with ZIKV and CHIKV or DENV. **Fig E**. Mapping of virus-specific 25–29 nts small RNAs produced in infected Ae. aegypti-derived AF5 cells, along the corresponding virus genome. **Fig F**. Characterisation of CHIKV-specific piRNA-like small RNAs in AF5 cells. **Fig G**. CHIKV and ZIKV infection in Ago2 knock out mosquito cells during single and co-infections. **Table A**. Primer sequences for qPCR. All sequences are shown 5’-3’. **Table B**. Small RNA sequencing data. **Table C**. Statistical analysis of dsRNA-based silencing experiments. **Table D**. Statistical analysis of infection in knockout cells. **Table E**. Comparison of ZIKV and DENV genome similarities of 21-18mers. **Table F**. Statistical analysis of luciferase data in knock out cells.
(DOCX)
