# Supplementary material for: The Aedes aegypti RNA interference response against Zika virus in the context of co-infection with dengue and chikungunya viruses
Source: PLoS Negl Trop Dis. 2023 Jul 13;17(7):e0011456. doi: 10.1371/journal.pntd.0011456 (PMC10343070; doi:10.1371/journal.pntd.0011456)
Supplement: S1 File — Fig A. Analysis of ZIKV-derived vsiRNAs from Ae. aegypti mosquitoes infected with ZIKV and CHIKV or DENV. Fig B. Mapping of virus-specific 25–29 nts small RNAs produced in infected mosquitoes, along the corresponding virus genome. Fig C. Characterisation of CHIKV-specific piRNA-like small RNAs in Ae. aegypti mosquitoes. Fig D. Analysis of ZIKV-derived vsiRNAs from AF5 cells infected with ZIKV and CHIKV or DENV. Fig E. Mapping of virus-specific 25–29 nts small RNAs produced in infected Ae. aegypti-derived AF5 cells, along the corresponding virus genome. Fig F. Characterisation of CHIKV-specific piRNA-like small RNAs in AF5 cells. Fig G. CHIKV and ZIKV infection in Ago2 knock out mosquito cells during single and co-infections. Table A. Primer sequences for qPCR. All sequences are shown 5’-3’. Table B. Small RNA sequencing data. Table C. Statistical analysis of dsRNA-based silencing experiments. Table D. Statistical analysis of infection in knockout cells. Table E. Comparison of ZIKV and DENV genome similarities of 21-18mers. Table F. Statistical analysis of luciferase data in knock out cells. (DOCX) [file pntd.0011456.s001.docx]

# Supplementary data, S1

**Fig A. Analysis of ZIKV-derived vsiRNAs from Ae. aegypti mosquitoes infected with ZIKV and CHIKV or DENV.** (A) To determine the differences in ZIKV-derived vsiRNAs during CHIV or DENV co-infection, relative mean mapped reads per million (RPM) to the genome (left) and anti-genome (right) were tested for linearity showing linear association between mapped reads with corresponding r^2^ values showing a relatively higher positive relationship for reads mapping to the genome versus the anti-genome. (B) To evaluate the abundance of ZIKV-derived vsiRNAs, relative mapped reads in RPM were expressed as log2 and depicted as a scatter plot with each quadrant showing either relative increase (arrow up) or decrease (arrow down) of mapped reads during co-infection with CHIKV or DENV revealing. No distinct scattering was observed with much of the reads centrally positioned except for some notable genome positions indicated by the numbers.





#
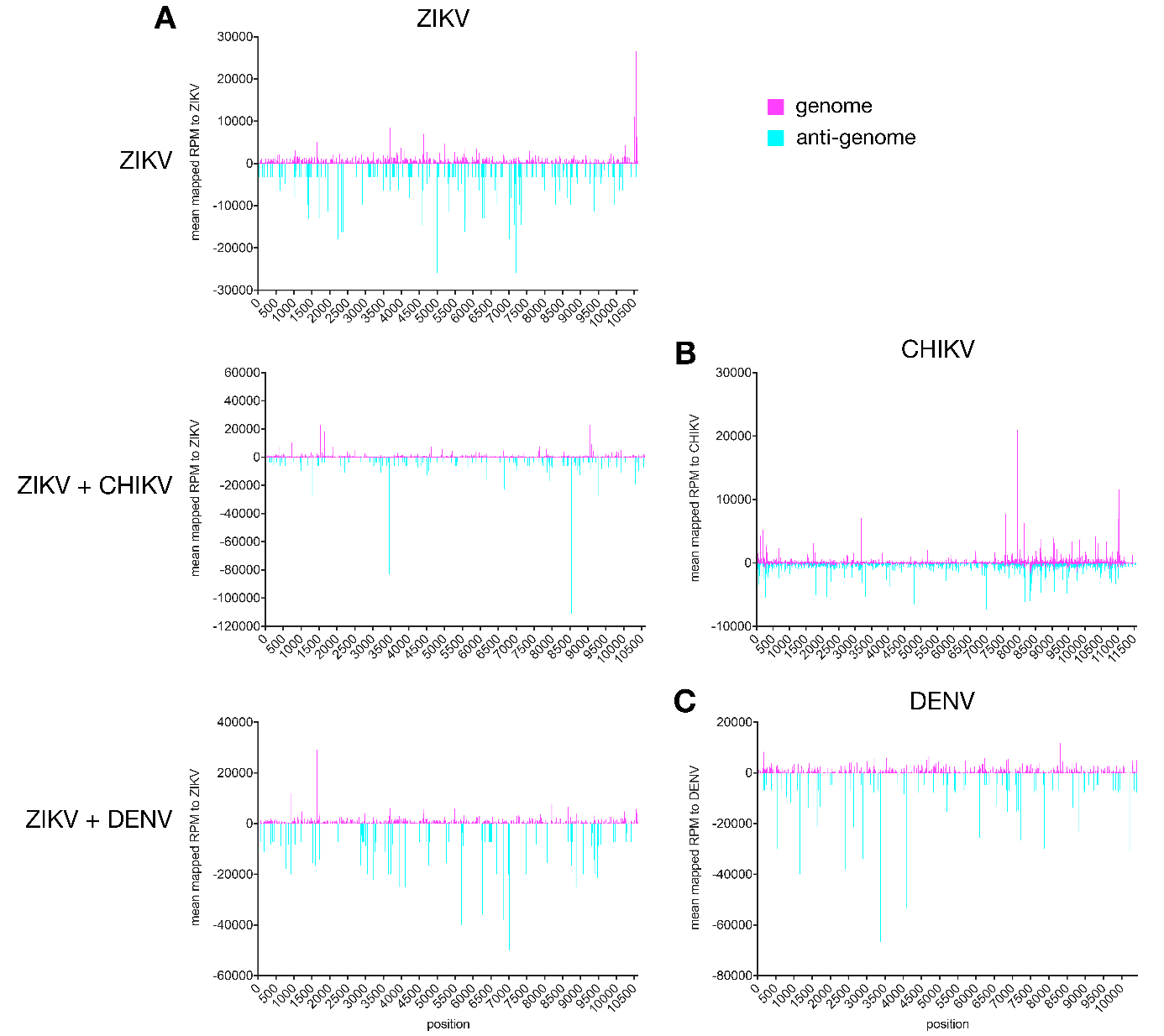


**Fig B. Mapping of virus-specific 25-29 nts small RNAs produced in infected mosquitoes, along the corresponding virus genome.** ZIKV (A) either single infection or co-infection with CHIKV (B) or DENV (C). The y axis shows the number of reads to the respective viral genome and anti-genome. An example of 2 (ZIKV+CHIKV) or 3 (ZIKV+DENV) repeats is shown.

**
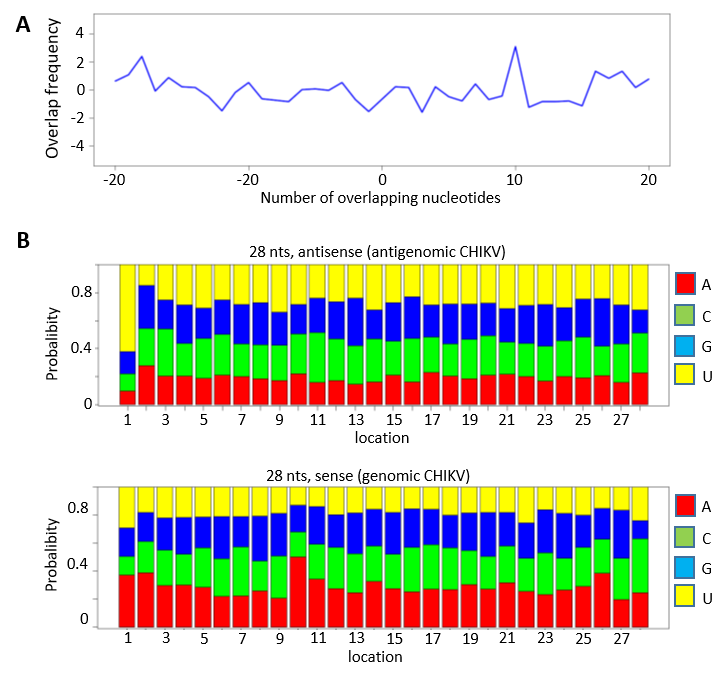
**

**Fig C**. **Characterisation of CHIKV-specific piRNA-like small RNAs in Ae. aegypti mosquitoes.** (A) Overlap frequencies of sense and antisense CHIKV-specific small RNAs. (B) Sequence logo plots showing the sequence bias in various positions of CHIKV-specific small RNAs for antigenomic (top) and genomic (bottom) small RNAs. Small RNAs with 28-nt in lengths were used as representative for the 25-30 nt long CHIKV-specific piRNAs. The data shown are representative results from two independent experiments.





**Fig D. Analysis of ZIKV-derived vsiRNAs from AF5 cells infected with ZIKV and CHIKV or DENV.** (A) To determine the differences in ZIKV-derived vsiRNAs during CHIV or DENV co-infection, relative mean mapped reads per million (RPM) to the genome (left) and anti-genome (right) were tested for linearity showing linear association between mapped reads with corresponding r^2^ values showing a low relationship for reads mapping to the genome versus the anti-genome. (B) To evaluate the abundance of ZIKV-derived vsiRNAs, relative mapped reads in RPM were expressed as log2 and depicted as a scatter plot with each quadrant showing either relative increase (arrow up) or decrease (arrow down) of mapped reads during co-infection with CHIKV or DENV revealing. No distinct scattering was observed with when comparing the reads mapping to the genome and anti-genome.

#
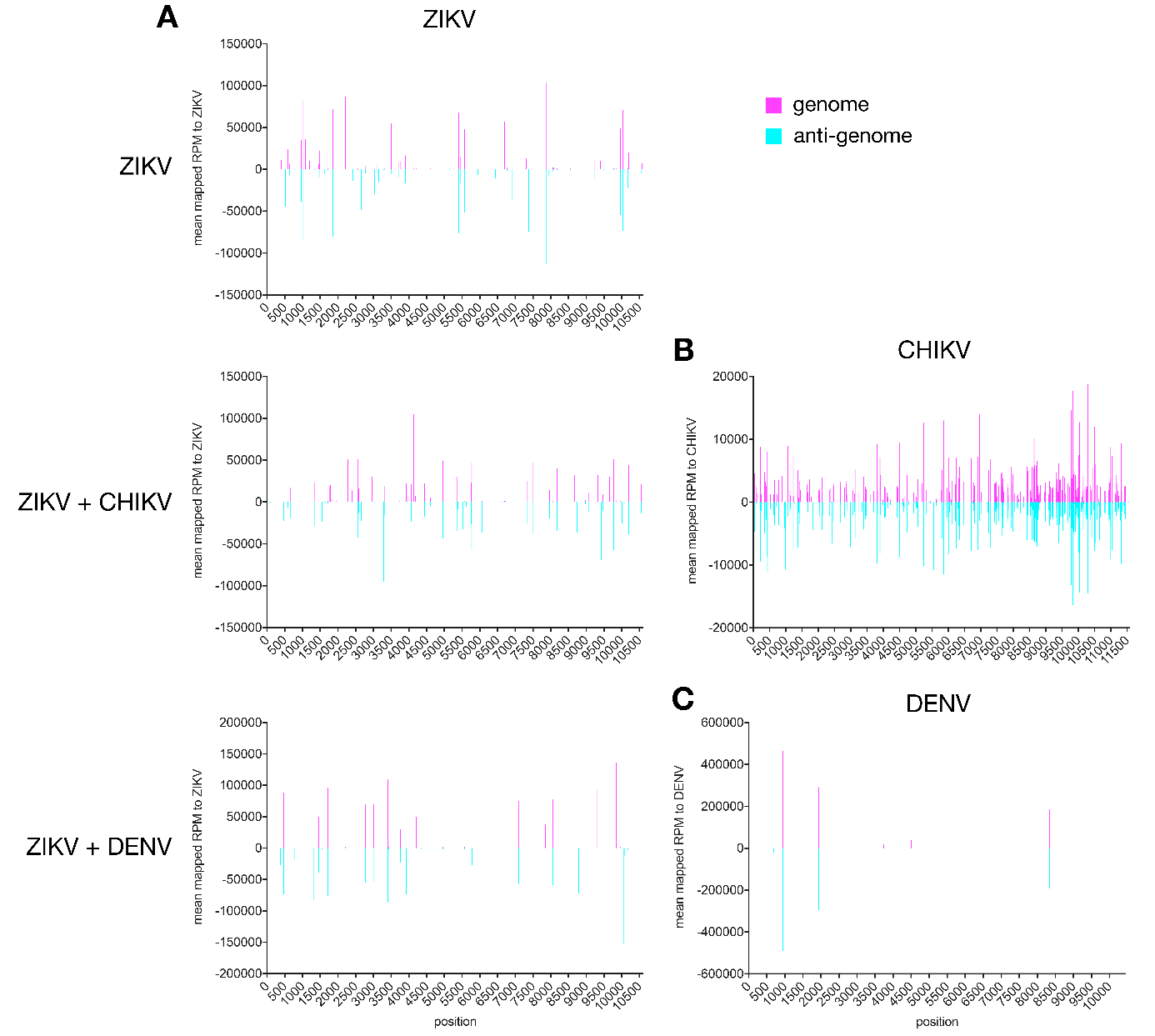


**Fig E. Mapping of virus-specific 25-29 nts small RNAs produced in infected Ae. aegypti-derived AF5 cells, along the corresponding virus genome.** ZIKV (A) either single infection or co-infection with CHIKV (B) or DENV (C). The y axis shows the number of reads to the respective viral genome and anti-genome. An example of 2 (ZIKV+CHIKV) repeats is shown.

**
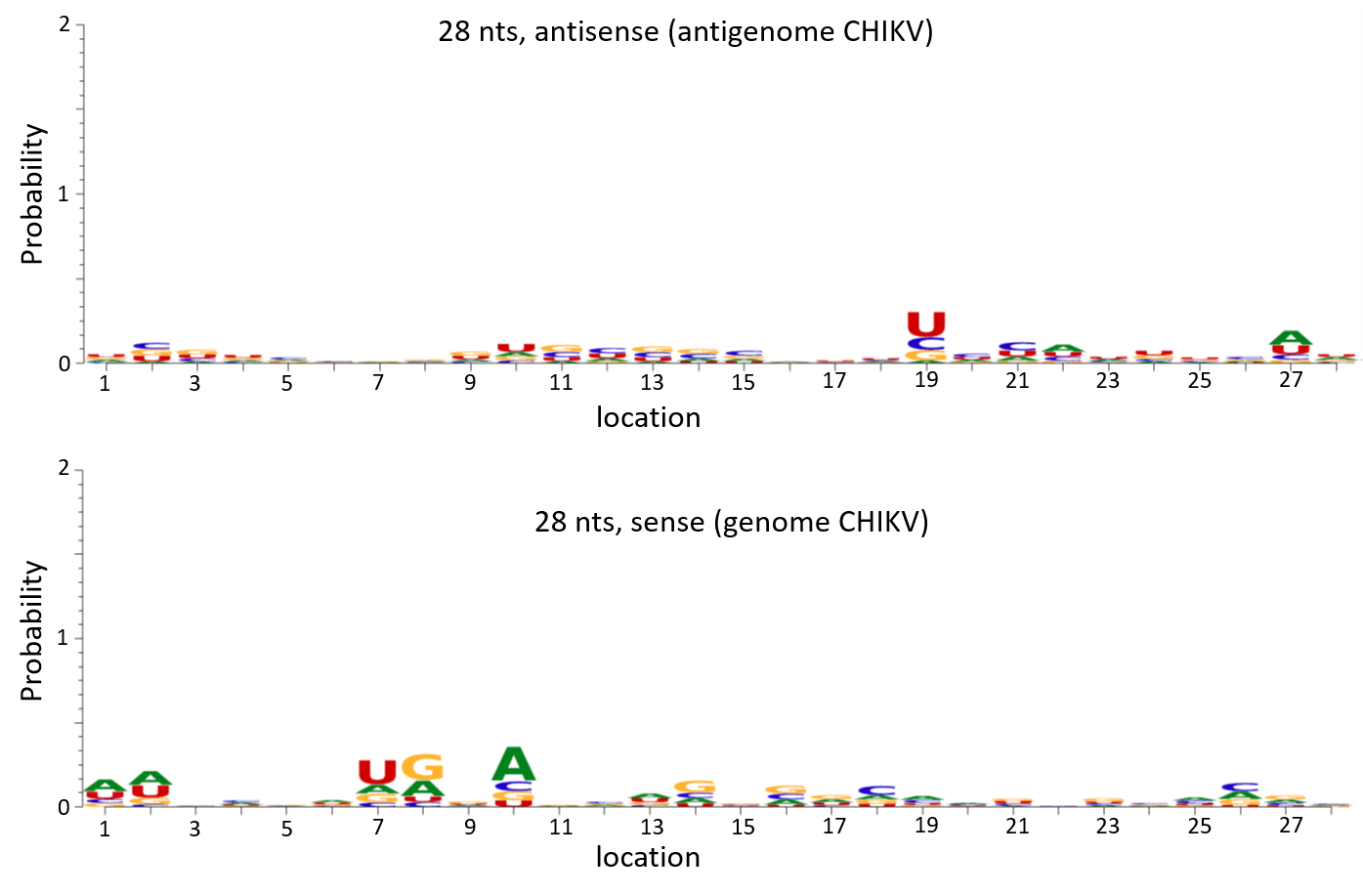
**

**Fig F**. **Characterisation of CHIKV-specific piRNA-like small RNAs in AF5 cells.**

Sequence logo plots showing the sequence bias in various positions of CHIKV-specific small RNAs for antigenomic (top) and genomic (bottom) small RNAs. Small RNAs with 28-nt in lengths were used as representative for the 25-30 nt long CHIKV-specific piRNAs. The data shown are representative results from two independent experiments.


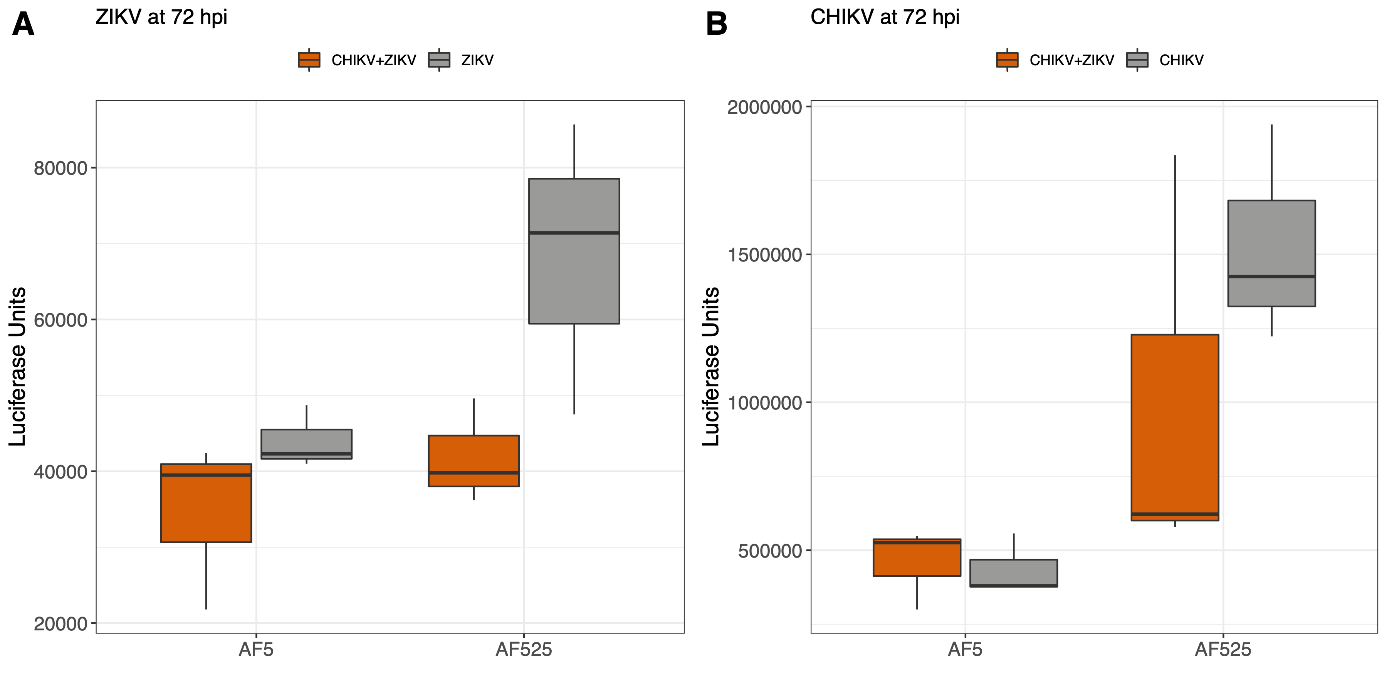


**Fig G. CHIKV and ZIKV infection in Ago2 knock out mosquito cells during single and co-infections*.***

*Ae. aegypti-derived AF525 (Ago2 deficient) or parental AF5 cells were infected either single (grey) with a Brazilian derived ZIKV expressing nanoluciferase (ZIKV-Nluc)(1) or with CHIKV expressing Firefly luciferase (CHIKV-2SG-FFluc)(2) or co-infected (ZIKV+CHIKV: orange) (MOI1). Firefly luciferase and Nanoluciferase (A: ZIKV, Nanoluciferase B: CHIKV, Firefly luciferase) at 72 hpi was determined by corresponding luciferase assays. Luciferase units are shown as the median with min and max values from five independent experiments performed in technical triplicates.*

**Table A.** Primer sequences for qPCR. All sequences are shown 5’-3’.

| Target | Forward/reverse primer |
| --- | --- |
| CHIKV | GCCTTTGCCGTTACGCTATG/  ATTCCCTTGGACTTACGCGC |
| ZIKV (3) | GTTGTCGCTGCTGAAATGGA/  GGGGACTCTGATTGGCTGTA |
| DENV | TGGCTGAAATTGCGTGACTC/  GCCAACTTCCAGGTTTCGTT |
| S7 (4) | CCAGGCTATCCTGGAGTTG/  GACGTGCTTGCCGGAGAAC |

**Table B.** Small RNA sequencing data.

| **Small RNA from infected *Ae. aegypti* mosquitoes** | | | | | | | | |
| --- | --- | --- | --- | --- | --- | --- | --- | --- |
| ***Sample*** | ***Total clean reads*** | ***ZIKV 21 nt %*** | ***ZIKV 25-29 nt %*** | ***CHIKV 21 nt %*** | ***CHIKV 25-29 nt %*** | ***DENV 21 nt %*** | ***DENV 25-29 nt %*** | ***Relative Ct values*** |
| ZIKV  (3x mosq) | 622816225 | 0,0121 | 0,0007 |  |  |  |  | 7,2 |
| ZIKV  (1x mosq.) | 534361513 | 0,0204 | 0,0009 |  |  |  |  | 21,7 |
| ZIKV+CHIKV  (3x mosq.) | 718663178 | 0,0024 | 0,0007 | 0,0142 | 0,0019 |  |  | ZIKV: 3  CHIKV: 17,6 |
| ZIKV+CHIKV  (1x mosq.) | 630571867 | 0,0017 | 0,0027 | 0,0170 | 0,0028 |  |  | ZIKV: 2,6  CHIKV: 26,9 |
| ZIKV+CHIKV  (2x mosq.) | 559232398 | 0,0069 | 0,0007 | 0,0161 | 0,0052 |  |  | ZIKV: 8,9  CHIKV: 83,1 |
| ZIKV+CHIKV  (2x mosq.) | 471629863 | 0,0012 | 0,0001 | 0,0542 | 0,0032 |  |  | ZIKV: 1,2  CHIKV: 126 |
| ZIKV+DENV  (1x mosq.) | 730298151 | 0,0003 | 0,0001 |  |  | 0,0003 | 0,0002 | ZIKV: 0,3  DENV: 0,02 |
| ZIKV+DENV  (1x mosq.) | 704431819 | 0,0036 | 0,0002 |  |  | 0,0009 | 0,0006 | ZIKV: 2,5  DENV: 0,7 |
| ZIKV+DENV  (3x mosq.) | 557809541 | 0,0006 | 0,0001 |  |  | 0,0002 | 0,0002 | ZIKV: 0,9  DENV: 1,9 |
| ZIKV+DENV  (2x mosq.) | 606284258 | 0,0038 | 0,0003 |  |  | 0,0010 | 0,0001 | ZIKV: 7,1  DENV: 0,4 |
| ZIKV+DENV  (2x mosq.) | 527129416 | 0,0018 | 0,0002 |  |  | 0,0005 | 0,0002 | ZIKV: 3,7  DENV: 0,3 |
| **Small RNA from infected *Ae. aegypti*-derived cells** | | | | | | | |  |
| **Sample** | **Total clean reads** | **ZIKV 21 nt %** | **ZIKV 25-29 nt %** | **CHIKV 21 nt %** | **CHIKV 25-29 nt %** | **DENV 21 nt %** | **DENV 25-29 nt %** |  |
| ZIKV | 29092192 | 0,1485 | 0,0037 |  |  |  |  |  |
| ZIKV | 22336862 | 0,1865 | 0,0059 |  |  |  |  |  |
| ZIKV+CHIKV | 38168298 | 0,1052 | 0,0023 | 0,2037 | 0,0335 |  |  |  |
| ZIKV+CHIKV | 30729256 | 0,1276 | 0,0041 | 0,2398 | 0,0375 |  |  |  |
| ZIKV+DENV | 33926096 | 0,0722 | 0,0032 |  |  | 0,0049 | 0,0006 |  |

**Table C.** Statistical analysis of dsRNA-based silencing experiments. Analyses were conducted first in the complete dataset (noted as general) and in the subsets based on the infection (ZIKV+DENV, ZIKV+CHIKV, ZIKV single, DENV single)

| **ZIKV general** | | **KWχ²** | **P** | **df** |
| --- | --- | --- | --- | --- |
|  | infection type (ZIKV single, co-infection with DENV, co-infection with CHIKV) | 0.63 | 0.731 | 2 |
|  | target (Ago2, Ago3, Piwi4, Piwi5, Piwi6, eGFP) | 39.04 | 2.33e-07 | 5 |
|  | Piwi4 KD vs eGFP (dunns test with Bonferroni correction) |  | 0.0009152592 |  |
| **ZIKV+DENV** | | **KWχ²** | **P** | **df** |
|  | target (Ago2, Ago3, Piwi4, Piwi5, Piwi6, eGFP) | 21.98 | 0.0005285 | 5 |
|  | Piwi4 vs eGFP(dunns test with Bonferroni correction) |  | 0.04893699 |  |
| **ZIKV single** | | **F** | **p** | **ds** |
|  | target (Ago2, Ago3, Piwi4, Piwi5, Piwi6, eGFP) | 11 | 0.0005285 | 5 |
|  | Piwi4 KD vs eGFP (dunns test with Bonferroni correction) |  | 0.0043 |  |
| **ZIKV+CHIKV** | | **F** | **p** | **df** |
|  | target (Ago2, Ago3, Piwi4, Piwi5, Piwi6, eGFP) | 6.158 | 0.00473 | 5 |
|  | Piwi4 KD vs eGFP (dunns test with Bonferroni correction) |  | 0.088 |  |
| **CHIKV** | | | | |
| ***CHIKV general*** | | ***KWχ²*** | ***P*** | ***df*** |
|  | infection type (ZIKV co-infection or single infection) | 0.049277 | 0.8243 | 1 |
|  | target (Ago2, Ago3, Piwi4, Piwi5, Piwi6, eGFP) | 19.973 | 0.001264 | 5 |
|  | Ago2 KD vs eGFP (dunns test with Bonferroni correction) |  | 0.008242526 |  |
| **DENV** | | | | |
| ***DENV general*** | | ***KWχ²*** | ***P*** | ***df*** |
|  | target (Ago2, Ago3, Piwi4, Piwi5, Piwi6, eGFP) | 22.399 | 0.0004396 | 5 |
|  | infection type (ZIKV co-infection or single infection) | 50.716 | 0.02432 | 1 |
| ***DENV single*** | | ***KWχ²*** | ***P*** | ***df*** |
|  | target (Ago2, Ago3, Piwi4, Piwi5, Piwi6, eGFP) | 16.78 | 0.004946 | 5 |
|  | Ago2 KD vs eGFP |  | 0.0007225399 |  |
|  | Ago3 KD vs eGFP |  | 0.0454770843 |  |
|  | PIWI6 KD vs eGFP |  | 0.0090084160 |  |
| ***DENV+ZIKV*** | | ***KWχ²*** | ***P*** | ***df*** |
|  | target (Ago2, Ago3, Piwi4, Piwi5, Piwi6, eGFP) | 7.8822 | 0.1663 | 5 |
|  |  |  |  |  |

**Table D.** Statistical analysis of infection in knockout cells

| **ZIKV** | | | | |
| --- | --- | --- | --- | --- |
| ***ANOVA*** | ***df*** | ***Sum Sq*** | ***F value*** | ***Pr (>F)*** |
| Cell (AF5 versus AF525) | 1 | 0.06241 | 16.968 | 0.00104** |
| Virus (ZIKV single, ZIKV+DENV, ZIKV+CHIKV) | 2 | 0.00460 | 0.625 | 0.54962 |
| Residuals | 14 | 0.05149 |  |  |
| ***Welch Two Sample t-test*** | ***df*** | ***t*** |  | ***p*** |
| ZIKV single (AF5 versus AF525) | 3.8892 | 1.7073 |  | 0.165 |
| ZIKV+CHIKV (AF5 versus AF525) | 2.5848 | 5.7885 |  | 0.01527 |
| ZIKV+DENV (AF5 versus AF525) | 3.9125 | 2.7379 |  | 0.05329 |
| **DENV** | | | | |
| ***ANOVA*** | ***df*** | ***Sum Sq*** | ***F value*** | ***Pr (>F)*** |
| Cell (AF5 versus AF525) | 1 | 0.4688 | 15.781 | 0.00324** |
| Virus (ZIKV single, ZIKV+DENV, ZIKV+CHIKV) | 1 | 0.1053 | 3.543 | 0.09245 |
| Residuals | 9 | 0.2674 |  |  |
| ***Welch Two Sample t-test*** | ***df*** | ***t*** |  | ***p*** |
| DENV single (AF5 versus AF525) | 2.2159 | 2.6123 |  | 0.1086 |
| DENV+ZIKV (AF5 versus AF525) | 3.379 | 3.049 |  | 0.04749 |
| **CHIKV** | | | | |
| ***Welch Two Sample t-test*** | ***df*** | ***t*** | ***p*** |  |
| Cell (AF5 versus AF525) | 2 | -2,9033 | 0.101 |  |

**Table E.** Comparison of ZIKV and DENV genome similarities of 21-18mers. HSP, high similarity pair)

**Table F.** Statistical analysis of luciferase data in knock out cells

| **ZIKV** | | **KWχ²** | **P** | **df** |
| --- | --- | --- | --- | --- |
|  | Cells in general (AF5 versus AF525) | 1.641 | 0.2002 | 1 |
|  | Infection (single versus ZIKV+CHIKV) | 3.6923 | 0.05466 | 1 |
| ***Welch Two Sample t-test*** |  | ***t*** | ***p*** | **df** |
|  | Cells only single infection (AF5 versus AF525) |  | 0.3901 | 4 |
| **CHIKV** | | **KWχ²** | **P** | **df** |
|  | Cells in general (AF5 versus AF525) | 8.3077 | 0.003948 | 1 |
|  | Infection (single versus ZIKV+CHIKV) | 0.23077 | 0.631 | 1 |
| ***Welch Two Sample t-test*** |  | ***t*** | ***p*** | **df** |
|  | Cells, only single infection (AF5 versus AF525) | -4.9304 | 0.007871 | 4 |

References:

1. Mutso M, Saul S, Rausalu K, Susova O, Zusinaite E, Mahalingam S, et al. Reverse genetic system, genetically stable reporter viruses and packaged subgenomic replicon based on a Brazilian Zika virus isolate. The Journal of general virology. 2017;98(11):2712-24.

2. Pohjala L, Utt A, Varjak M, Lulla A, Merits A, Ahola T, et al. Inhibitors of alphavirus entry and replication identified with a stable Chikungunya replicon cell line and virus-based assays. PloS one. 2011;6(12):e28923.

3. Varjak M, Donald CL, Mottram TJ, Sreenu VB, Merits A, Maringer K, et al. Characterization of the Zika virus induced small RNA response in Aedes aegypti cells. PLoS neglected tropical diseases. 2017;11(10):e0006010.

4. McFarlane M, Arias-Goeta C, Martin E, O'Hara Z, Lulla A, Mousson L, et al. Characterization of Aedes aegypti innate-immune pathways that limit Chikungunya virus replication. PLoS neglected tropical diseases. 2014;8(7):e2994.
